# Time Trends in Ischemic Stroke among Type 2 Diabetic and Non-Diabetic Patients: Analysis of the Spanish National Hospital Discharge Data (2003-2012)

**DOI:** 10.1371/journal.pone.0145535

**Published:** 2015-12-29

**Authors:** Nuria Muñoz-Rivas, Manuel Méndez-Bailón, Valentín Hernández-Barrera, José Ma de Miguel-Yanes, Rodrigo Jiménez-García, Jesús Esteban-Hernández, Isabel Jiménez-Trujillo, Alejandro Alvaro-Meca, Pilar Carrasco-Garrido, Javier de Miguel-Díez, Ana López-de-Andrés

**Affiliations:** 1 Medicine Department, Hospital Universitario Infanta Leonor, Madrid, Spain; 2 Medicine Department, Hospital Clínico San Carlos, Madrid, Comunidad de Madrid, Spain; 3 Preventive Medicine and Public Health Teaching and Research Unit, Health Sciences Faculty, Rey Juan Carlos University, Alcorcon, Comunidad de Madrid, Spain; 4 Medicine Department, Hospital Gregorio Marañon, Comunidad de Madrid, Spain; 5 Pneumology Department, Hospital General Universitario Gregorio Marañón, Universidad Complutense de Madrid, Madrid, Spain; University of Oklahoma Health Sciences Center, UNITED STATES

## Abstract

**Background:**

Type 2 Diabetes (T2DM) is the most rapidly increasing risk factor for ischemic stroke. We aimed to compare trends in outcomes for ischemic stroke in people with or without diabetes in Spain between 2003 and 2012.

**Methods:**

We selected all patients hospitalized for ischemic stroke using national hospital discharge data. We evaluated annual incident rates stratified by T2DM status. We analyzed trends in the use of diagnostic and therapeutic procedures, patient comorbidities, and in-hospital outcomes. We calculated in-hospital mortality (IHM), length of hospital stay (LOHS) and readmission rate in one month after discharge. Time trend on the incidence of hospitalization was estimated fitting Poisson regression models by sex and diabetes variables. In-hospital mortality was analyzed using logistic regression models separate for men and women. LOHS were compared with ANOVA or Kruskal-Wallis when necessary.

**Results:**

We identified a total of 423,475 discharges of patients (221,418 men and 202,057 women) admitted with ischemic stroke as primary diagnosis. Patients with T2DM accounted for 30.9% of total. The estimated incidence rates of discharges increased significantly in all groups. The incidence of hospitalization due to stroke (with ICD9 codes for stroke as main diagnosis at discharge) was higher among those with than those without diabetes in all the years studied. T2DM was positively associated with ischemic stroke with an adjusted incidence rate ratio (IRR) of 2.27 (95% CI 2.24–2.29) for men and 2.15 (95%CI 2.13–2.17) for women. Over the 10 year period LOHS decreased significantly in men and women with and without diabetes. Readmission rate remained stable in diabetic and non diabetic men (around 5%) while slightly increased in women with and without diabetes. We observed a significant increase in the use of fibrinolysis from 2002–2013. IHM was positively associated with older age in all groups, with Charlson Comorbidity Index > 3 and atrial fibrillation as risk factors. The IHM did not change significantly over time among T2DM men and women ranging from 9.25% to 10.56% and from 13.21% to 14.86%, respectively; neither did among non-diabetic women. However, in men without T2DM IHM decreased significantly over time. Diabetes was associated to higher IHM only in women (OR 1.07; 95% CI, 1.05–1.11).

**Conclusions:**

Our national data show that incidence rate of ischemic stroke hospitalization increased significantly during the period of study (2003–2012). People with T2DM have more than double the risk of ischemic stroke after adjusting for other risk factors. Women with T2DM had poorer outcomes- IHM and readmission rates- than diabetic men. Diabetes was an independent factor for IHM only in women.

## Background

Strokes or cerebrovascular accidents (CVA) represent one of the most important public health problems particularly in industrialized countries [1]. Stroke is the second leading cause of death worldwide and non fatal stroke is the second leading cause of long-term disability in high-income countries [2]. Ischemic strokes represent around 80–87 percent of all CVA [3].

Type 2 Diabetes (T2DM) and ischemic stroke often come up together. Diabetes causes atherosclerotic changes in the heart and the cerebropetal arteries and is associated with different subtypes of ischemic stroke, including lacunar, large artery occlusive and thromboembolic strokes [4]. T2DM is a significant risk factor for stroke conferring two to three times increase in relative risk [5,6] and it is also associated to an increased risk of mortality [7]. Its independent effect on incidence of ischemic stroke is well stablished [8].

In Spain, CVA are the second leading cause of mortality in general population, and the first in women [9], specially affecting the elderly [10]. Stroke incidence has been evaluated in several studies in our country with a great variability between them [11] and most of studies included both, ischemic and hemorrhagic subtypes. Incidence rates fluctuate between 120 and 350 cases per 100.000 people/year [12,13]. The IBERICTUS study that included transient ischemic attack, ischemic and hemorrhagic stroke estimates 187 new cases per 100.000 people, 150 of them were ischemic ones, with the greatest impact in over 85 years old group [14]. The prevalence of diabetes is constantly increasing and elderly people account for nearly 40% of the diabetic population [15]. Moreover, nearly 20% of diabetic people die from CVA [16]. For the reasons above, it can be expected that stroke will continue to be a health concern.

However, despite T2DM has become an increasingly common disease, a recent review showed a 42% decrease in stroke incidence rates in high-income countries over the past four decades [17]. Limited information is available regarding the trend in ischemic stroke incidence in Spain. To reliably estimate the burden of T2DM associated diseases for health care planning, a better understanding of how T2DM affects the risk of stroke in clinically important subgroups is needed.

The aim of this study was to compare trends in the incidence rates and outcomes of ischemic stroke among hospitalized men and women with and without type 2 diabetes between 2003 and 2012 in Spain using national hospital discharge data. In particular, we analyzed trends in the use of diagnostic and therapeutic procedures such as fibrinolysis, patient comorbidities, and in-hospital outcomes- length of hospital stay (LOHS), readmissions rates and in-hospital mortality (IHM).

## Methods

This retrospective, observational study was conducted using the Spanish National Hospital Database (CMBD, *Conjunto Mínimo Básico de Datos*). This database is managed by the Spanish Ministry of Health, Social Services and Equality and compiles all public and private hospital data, hence covering more than 95% of hospital discharges [18]. The CMBD includes patient variables (sex, date of birth), admission date, discharge date, up to 14 discharge diagnoses, and up to 20 procedures performed during the hospital stay. The Spanish Ministry of Health, Social Services and Equality sets standards for record-keeping and performs periodic audits [18].

Disease and procedure criteria were defined according to the International Classification of Diseases-Ninth Revision, Clinical Modification (ICD-9-CM), which is used in the Spanish CMBD.

We selected all patients hospitalized for ischemic stroke (ICD-9-CM codes 434.01, 434.11 and 434.91) as the primary diagnosis between January 1, 2003 and December 31, 2012. Discharges were grouped by diabetes status as follows: type 2 diabetes (ICD-9-CM codes: 250.x0; 250.x2) and no diabetes. Patients with type 1 diabetes (ICD-9-MC codes: 250.x1; 250.x3) were excluded.

We calculated the incidence of discharge rates after ischemic stroke for men and women with and without type 2 diabetes per 100,000 inhabitants. We calculated yearly diabetes-specific incidence rates dividing the number of cases per year, sex, and age group by the corresponding number of people in that population group, using age- and sex-adjusted estimated prevalence of diabetes obtained from National Health Surveys conducted in 2003/4, 2006/7, 2009/10 and 2011/12 and data from Di@bet.es Study [19,20]. From 2001 till 2010, Spanish National Health Survey has been published every two or three years. So diabetic population for missing years (2005 and 2008) was estimated assuming that growth rate was the same thorough the period 2004–2010. We estimated rate fitting a linear regression model with population from years when NHS was available and we used this model to impute population for 2005 and 2008. We also calculated the yearly age- and sex-specific incidence rates for non-diabetic patients dividing the number of cases per year, sex, and age group by the corresponding number of people in that population group (excluding those with type 2 diabetes), according to data from the Spanish National Institute of Statistics, as reported on December 31 of each year [21].

Clinical characteristics included information on overall comorbidity at the time of diagnosis, which was assessed by calculating the Charlson Comorbidity Index (CCI). The index applies to 17 disease categories, the scores of which are added to obtain an overall score for each patient [22]. We divided patients into three categories: low index, which corresponds to patients with no previously recorded disease or with one disease category; medium index, patients with two categories; and high index, patients with three or more disease categories. To calculate the CCI we used 15 disease categories, excluding diabetes and stroke as described by Thomsen RW *et al*. [23].

We analyzed the presence of hypertension (ICD-9-CM codes: 401, 401.0, 401.1, 401.9) and atrial fibrillation (ICD-9-CM code: 427.31) in any diagnosis position during ischemic stroke hospitalization. Information on current smoking was also identified using ICD-9-CM codes: 305.1 and V1582.

We specifically identified the following diagnostic and therapeutic procedures: magnetic resonance (ICD-9-CM code: 88.91), computed tomography angiography (CAT) (ICD-9-CM code: 87.03), doppler echocardiography (ICD-9-CM code: 88.71), mechanical ventilation (ICD-9-CM codes: 96.7, 96.70, 96.71, 96.72), parenteral nutrition (ICD-9-CM codes: 99.15) and fibrinolysis (ICD-9-CM codes: 99.10).

Patient readmissions were defined as inpatient re-hospitalization within 30 days after discharge (30-day readmission) regardless of that new hospitalization was related to previous one or not. So that, case definition in this study included patients with ICD9 codes for stroke in the primary position, no matter they were readmissions or not.

The median LOHS and the proportion of patients that died during the hospital admission (IHM) were also estimated for each year studied.

### Statistical analysis

A descriptive statistical analysis was performed for all continuous variables and categories by stratifying discharges for ischemic stroke according to diabetes status and sex. According to type and distribution of the variables, they were described using proportions, means with standard deviations or medians with interquartile ranges (LOHS). Bivariate analyses of the changes in variables according to year included Poisson regression (incidence by year of discharge), Pearson's chi-square (proportions), ANOVA (means) and Kruskal-Wallis test (medians). We fitted separate multivariate Poisson regression models for men and women with and without type 2 diabetes with age, CCI, hypertension, atrial fibrillation, smoking, diagnostic and therapeutic procedures, readmission and year of discharge as independent variables. So that estimates correspond to Incidence Rate Ratio (IRR) with their 95% confidence intervals. The inclusion of year of discharge allow us to estimate the average yearly rate of change.

A global model including the same variables and diabetes status was also conducted to assess the adjusted effect of diabetes in the incidence for each sex.

In-hospital mortality was analyzed fitting separate logistic regression models for men and women, with death during the hospitalization as a binary outcome using the same independent variables for those with and without diabetes and for the entire population to assess the influence of diabetes on IHM. Estimates were Odds Ratios with their 95% confidence intervals. Statistical analyses were performed using Stata version 10.1 (Stata, College Station, Texas, USA). Statistical significance was set at p<0.05 (2-tailed).

### Ethics

Data confidentiality was maintained at all times in accordance with Spanish legislation. Patient identifiers were deleted before the database was provided to the authors in order to maintain patient anonymity. It is not possible to identify patients on individual levels, either in this article or in the database. Readmission variable is created by technical staff at the Ministry of Health using information from several variables (patient National Health System identification code, date of birth and sex). After that, database is anonymized prior to delivery for investigation purposes.

Given the anonymous and mandatory nature of the dataset, it was not necessary to obtain informed consent. The study protocol was approved by the ethics committee of the Universidad Rey Juan Carlos.

## Results

We identified a total of 423,475 discharges of patients (221,418 men and 202,057 women) admitted with ischemic stroke as primary diagnosis in Spain from 2003 to 2012. Patients with type 2 diabetes accounted for 30.9% of total (68,267 men and 62,591 women).

Table 1 shows the annual hospital discharges rates and clinical characteristic for men with an ischemic stroke discharge diagnosis according to diabetes status from 2003 to 2012.

**Table 1:** Incidences and clinical characteristics of hospital discharges due to ischemic stroke among men with and without type 2 diabetes in Spain, 2003–2012.

	2003	2004	2005	2006	2007	2008	2009	2010	2011	2012	Total
NO DIABETES											
N	12159	12345	12471	14791	15740	16692	16828	17293	17308	17524	153151
Incidence*	74.68	75.83	74.49	85.98	91.5	95.83	95.43	98.06	99.02	100.26	89.38
Age, mean (SD)*	70.74(12.51)	70.95(12.4)	71.09(12.76)	71.18(12.95)	71.19(13.08)	71.34(13.17)	71.28(13.28)	71.48(13.35)	71.10(13.50)	71.46(13.51)	71.21(13.11)
15–44 years n (%)*	433(3.56)	397(3.22)	480(3.85)	553(3.74)	620(3.94)	622(3.73)	653(3.88)	638(3.69)	687(3.97)	656(3.74)	5739(3.75)
45–54 years n (%)*	921(7.57)	938(7.6)	932(7.47)	1166(7.88)	1306(8.3)	1386(8.3)	1455(8.65)	1506(8.71)	1570(9.07)	1642(9.37)	12822(8.37)
55–64 years n (%)*	1805(14.84)	1989(16.11)	1914(15.35)	2255(15.25)	2380(15.12)	2632(15.77)	2586(15.37)	2704(15.64)	2790(16.12)	2682(15.3)	23737(15.5)
65–74 years n (%)*	3708(30.5)	3528(28.58)	3362(26.96)	3909(26.43)	3967(25.2)	4033(24.16)	3991(23.72)	4029(23.3)	3956(22.86)	3880(22.14)	38363(25.05)
75–84 years n (%)*	4013(33)	4140(33.54)	4366(35.01)	5032(34.02)	5380(34.18)	5598(33.54)	5704(33.9)	5681(32.85)	5686(32.85)	5832(33.28)	51432(33.58)
>84 years n (%)*	1279(10.52)	1353(10.96)	1417(11.36)	1876(12.68)	2087(13.26)	2421(14.5)	2439(14.49)	2735(15.82)	2619(15.13)	2832(16.16)	21058(13.75)
CCI 0–1, n (%)*	7303(60.06)	7288(59.04)	7415(59.46)	8889(60.1)	9600(60.99)	9768(58.52)	9790(58.18)	9833(56.86)	8895(51.39)	8736(49.85)	87517(57.14)
CCI 2, n (%)*	4682(38.51)	4881(39.54)	4894(39.24)	5683(38.42)	5923(37.63)	6596(39.52)	6740(40.05)	7089(40.99)	7979(46.1)	8315(47.45)	62782(40.99)
CCI ≥3, n (%)*	174(1.43)	176(1.43)	162(1.3)	219(1.48)	217(1.38)	328(1.97)	298(1.77)	371(2.15)	434(2.51)	473(2.7)	2852(1.86)
Atrial fibrillation, n (%)*	2447(20.13)	2485(20.13)	2591(20.78)	3025(20.45)	3137(19.93)	3619(21.68)	3640(21.63)	3926(22.7)	3993(23.07)	4128(23.56)	32991(21.54)
Hypertension n (%)*	5671(46.64)	5875(47.59)	6002(48.13)	7128(48.19)	7645(48.57)	8336(49.94)	8466(50.31)	8847(51.16)	8808(50.89)	8874(50.64)	75652(49.4)
Smoking n (%)*	3987(32.79)	4252(34.44)	4230(33.92)	5315(35.93)	5300(33.67)	5792(34.7)	6169(36.66)	6301(36.44)	6505(37.58)	6427(36.68)	54278(35.44)
DIABETES											
N	4771	5080	5375	6221	6995	7572	7684	7934	8268	8367	68267
Incidence*	492.26	524.15	505.69	537.87	604.79	640.24	635.69	656.37	581.94	588.91	580.69
Age, mean (SD)*	70.65(9.89)	70.93(10.05)	71.03(10.13)	71.26(10.39)	71.55(10.25)	71.54(10.54)	71.75(10.66)	71.92(10.86)	72.02(10.82)	72.12(10.75)	71.57(10.51)
15–44 years n (%)*	54(1.13)	41(0.81)	47(0.87)	58(0.93)	57(0.81)	70(0.92)	78(1.02)	89(1.12)	69(0.83)	74(0.88)	637(0.93)
45–54 years n (%)*	250(5.24)	302(5.94)	321(5.97)	380(6.11)	385(5.5)	482(6.37)	477(6.21)	501(6.31)	532(6.43)	483(5.77)	4113(6.02)
55–64 years n (%)*	874(18.32)	933(18.37)	980(18.23)	1095(17.6)	1268(18.13)	1336(17.64)	1330(17.31)	1363(17.18)	1408(17.03)	1464(17.5)	12051(17.65)
65–74 years n (%)*	1810(37.94)	1780(35.04)	1844(34.31)	2066(33.21)	2221(31.75)	2330(30.77)	2293(29.84)	2287(28.83)	2367(28.63)	2380(28.45)	21378(31.32)
75–84 years n (%)*	1478(30.98)	1674(32.95)	1826(33.97)	2135(34.32)	2477(35.41)	2675(35.33)	2754(35.84)	2833(35.71)	3014(36.45)	3018(36.07)	23884(34.99)
>84 years n (%)*	305(6.39)	350(6.89)	357(6.64)	487(7.83)	587(8.39)	679(8.97)	752(9.79)	861(10.85)	878(10.62)	948(11.33)	6204(9.09)
CCI 0–1, n (%)*	2807(58.83)	3001(59.07)	3100(57.67)	3673(59.04)	4130(59.04)	4303(56.83)	4398(57.24)	4269(53.81)	4039(48.85)	3930(46.97)	37650(55.15)
CCI 2, n (%)*	1902(39.87)	2017(39.7)	2182(40.6)	2458(39.51)	2755(39.39)	3151(41.61)	3107(40.43)	3511(44.25)	4018(48.6)	4173(49.87)	29274(42.88)
CCI ≥3, n (%)*	62(1.3)	62(1.22)	93(1.73)	90(1.45)	110(1.57)	118(1.56)	179(2.33)	154(1.94)	211(2.55)	264(3.16)	1343(1.97)
Atrial fibrillation n (%)*	730(15.3)	792(15.59)	935(17.4)	986(15.85)	1146(16.38)	1297(17.13)	1382(17.99)	1437(18.11)	1577(19.07)	1706(20.39)	11988(17.56)
Hypertension n (%)*	2723(57.07)	3089(60.81)	3278(60.99)	3883(62.42)	4435(63.4)	4800(63.39)	4985(64.88)	5132(64.68)	5353(64.74)	5443(65.05)	43121(63.17)
Smoking n (%)*	1445(30.29)	1571(30.93)	1804(33.56)	1990(31.99)	2257(32.27)	2530(33.41)	2644(34.41)	2805(35.35)	2922(35.34)	2949(35.25)	22917(33.57)

N: Number of discharges; Incidence: per 100,000 inhabitants; CCI (Charlson Comorbidity Index): Comorbidities included in the Charlson comorbidity index, except diabetes and stroke.

* P<0.05 (Comparison by year: Poisson regression model for incidence rates, ANOVA for means, Kruskal-Wallis for medians, Pearson´s chi-square for proportions).

Mean age was 71.57 years (SD, 10.51 years) in diabetic men and 71.21 years (SD, 13.11 years) in men without diabetes. Percentage of men with type 2 diabetes who have two or more coexisting conditions according to the CCI is slightly higher than that of non diabetic men (44.85% vs. 42.85%).

Smoking was also similar among men with type 2 diabetes (33.57%) than among non diabetic ones (35.47%). Hypertension was significantly more prevalent in diabetic men than in those without diabetes (63.17% and 49.4% respectively) but atrial fibrillation was more frequent (p<0.05) in non-diabetic men (21.54% and 17.56%, respectively).

The estimated incidence rate of discharges due to ischemic stroke in men with diabetes increases significantly from 492.26 cases per 100,000 diabetic men in 2003 to 588.91 cases in 2012. In men without diabetes, the incidence rate also increased significantly from 74.68 cases per 100,000 non-diabetic men in 2003 to 100.26 cases in 2012. Incidence rates of ischemic stroke were consistently higher among those with diabetes than those without diabetes in all the years studied.

At discharge, diabetic and non diabetic men hospitalized in 2012 are older, have more comorbidities and higher prevalence of smoking, hypertension and atrial fibrillation than patients hospitalized in 2003. Over the period study, 47.7% of all ischemic stroke discharges were women. Mean age for women with and without type 2 diabetes was 77.5 years. Women with type 2 diabetes had higher CCI values compared to those without diabetes (41.3% and 39.29% with two or more coexisting conditions, respectively).

Atrial fibrillation and smoking were more prevalent (34.28% and 5.53% vs. 30.14% and 2.82%) in women without diabetes. However hypertension was more prevalent in diabetic women (70.57% vs. 57.36%, p<0.05).

As can been seen in Table 2, incidence rate of hospitalization among women with type 2 diabetes increases significantly from 422.85 cases per 100,000 diabetic women in 2003 to 504.67 cases in 2012. Incidence rates also increased from 62.38 cases per 100,000 in 2003 to 92.25 cases in 2012 (p<0.05) among non diabetic women. As happened with men, rates were consistently higher in diabetic group.

**Table 2:** Incidences and clinical characteristics of hospital discharges due to ischemic stroke among women with and without type 2 diabetes in Spain, 2003–2012.

	2003	2004	2005	2006	2007	2008	2009	2010	2011	2012	Total
NO DIABETES											
N	10596	10609	10941	13122	14296	15114	15586	16077	16160	16965	139466
Incidence*	62.38	62.45	62.68	73.22	79.77	83.38	85.02	87.7	87.88	92.25	77.98
Age, mean (SD)*	76.68(12.08)	76.68(12.31)	76.9(12.42)	77.34(12.4)	77.34(12.62)	77.59(12.78)	78.01(12.51)	77.89(12.73)	78(12.94)	77.99(12.99)	77.53(12.63)
15–44 years n (%)*	292(2.76)	287(2.71)	312(2.85)	365(2.78)	386(2.7)	464(3.07)	406(2.6)	428(2.66)	450(2.78)	501(2.95)	3891(2.79)
45–54 years n (%)*	374(3.53)	380(3.58)	385(3.52)	459(3.5)	550(3.85)	574(3.8)	540(3.46)	648(4.03)	679(4.2)	688(4.06)	5277(3.78)
55–64 years n (%)*	632(5.96)	772(7.28)	740(6.76)	843(6.42)	941(6.58)	946(6.26)	1034(6.63)	1029(6.4)	1062(6.57)	1118(6.59)	9117(6.54)
65–74 years n (%)*	2181(20.58)	2063(19.45)	1988(18.17)	2320(17.68)	2412(16.87)	2404(15.91)	2402(15.41)	2509(15.61)	2358(14.59)	2498(14.72)	23135(16.59)
75–84 years n (%)*	4407(41.59)	4310(40.63)	4594(41.99)	5303(40.41)	5711(39.95)	5935(39.27)	6166(39.56)	6121(38.07)	6084(37.65)	6278(37.01)	54909(39.37)
>84 years n (%)*	2710(25.58)	2797(26.36)	2922(26.71)	3832(29.2)	4296(30.05)	4791(31.7)	5038(32.32)	5342(33.23)	5527(34.2)	5882(34.67)	43137(30.93)
CCI 0–1, n (%)*	6743(63.64)	6777(63.88)	7099(64.88)	8462(64.49)	9410(65.82)	9563(63.27)	9747(62.54)	9745(60.61)	8637(53.45)	8496(50.08)	84679(60.72)
CCI 2, n (%)*	3784(35.71)	3766(35.5)	3777(34.52)	4571(34.83)	4809(33.64)	5426(35.9)	5681(36.45)	6157(38.3)	7269(44.98)	8155(48.07)	53395(38.29)
CCI ≥3, n (%)*	69(0.65)	66(0.62)	65(0.59)	89(0.68)	77(0.54)	125(0.83)	158(1.01)	175(1.09)	254(1.57)	314(1.85)	1392(1)
Atrial fibrillation n (%)*	3534(33.35)	3606(33.99)	3764(34.4)	4365(33.26)	4659(32.59)	5104(33.77)	5331(34.2)	5669(35.26)	5684(35.17)	6096(35.93)	47812(34.28)
Hypertension n (%)*	5774(54.49)	5965(56.23)	6224(56.89)	7496(57.13)	8129(56.86)	8720(57.69)	9152(58.72)	9462(58.85)	9362(57.93)	9707(57.22)	79991(57.36)
Smoking n (%)*	407(3.84)	449(4.23)	476(4.35)	635(4.84)	679(4.75)	828(5.48)	894(5.74)	997(6.2)	1110(6.87)	1231(7.26)	7706(5.53)
DIABETES											
N	4755	4798	5060	5953	6214	6918	7251	7156	7231	7255	62591
Incidence*	422.85	426.67	445.15	518.17	540.89	572.76	572.37	564.88	503	504.67	508.87
Age, mean (SD)*	75.93(9.29)	76.36(9.41)	76.25(9.35)	77.22(9.49)	77.62(9.28)	77.93(9.47)	77.96(9.65)	77.99(9.61)	78.25(9.88)	78.25(9.63)	77.51(9.56)
15–44 years n (%)*	24(0.5)	25(0.52)	25(0.49)	27(0.45)	24(0.39)	29(0.42)	28(0.39)	32(0.45)	37(0.51)	29(0.4)	280(0.45)
45–54 years n (%)*	95(2)	96(2)	111(2.19)	127(2.13)	128(2.06)	115(1.66)	147(2.03)	137(1.91)	179(2.48)	153(2.11)	1288(2.06)
55–64 years n (%)*	380(7.99)	373(7.77)	412(8.14)	406(6.82)	394(6.34)	483(6.98)	499(6.88)	488(6.82)	481(6.65)	478(6.59)	4394(7.02)
65–74 years n (%)*	1373(28.87)	1316(27.43)	1319(26.07)	1419(23.84)	1412(22.72)	1471(21.26)	1501(20.7)	1457(20.36)	1317(18.21)	1387(19.12)	13972(22.32)
75–84 years n (%)*	2066(43.45)	2091(43.58)	2302(45.49)	2679(45)	2845(45.78)	3102(44.84)	3204(44.19)	3259(45.54)	3193(44.16)	3294(45.4)	28035(44.79)
>84 years n (%)*	817(17.18)	897(18.7)	891(17.61)	1295(21.75)	1411(22.71)	1718(24.83)	1872(25.82)	1783(24.92)	2024(27.99)	1914(26.38)	14622(23.36)
CCI 0–1, n (%)*	3015(63.41)	3061(63.8)	3200(63.24)	3752(63.03)	3920(63.08)	4151(60)	4364(60.18)	4159(58.12)	3577(49.47)	3538(48.77)	36737(58.69)
CCI 2, n (%)*	1707(35.9)	1693(35.29)	1827(36.11)	2155(36.2)	2230(35.89)	2681(38.75)	2811(38.77)	2906(40.61)	3523(48.72)	3568(49.18)	25101(40.1)
CCI ≥3, n (%)*	33(0.69)	44(0.92)	33(0.65)	46(0.77)	64(1.03)	86(1.24)	76(1.05)	91(1.27)	131(1.81)	149(2.05)	753(1.2)
Atrial fibrillation n (%)*	1284(27)	1310(27.3)	1510(29.84)	1751(29.41)	1824(29.35)	2037(29.44)	2256(31.11)	2262(31.61)	2299(31.79)	2334(32.17)	18867(30.14)
Hypertension n (%)*	3241(68.16)	3279(68.34)	3595(71.05)	4281(71.91)	4439(71.44)	4906(70.92)	5103(70.38)	5070(70.85)	5121(70.82)	5137(70.81)	44172(70.57)
Smoking n (%)*	93(1.96)	86(1.79)	99(1.96)	146(2.45)	146(2.35)	174(2.52)	217(2.99)	251(3.51)	269(3.72)	297(4.09)	1778(2.84)

N: Number of discharges; Incidence: per 100,000 inhabitants; CCI (Charlson Comorbidity Index): Comorbidities included in the Charlson comorbidity index, except diabetes and stroke.

* P<0.05 (Comparison by year: Poisson regression model for incidence rates, ANOVA for means, Kruskal-Wallis for medians, Pearson´s chi-square for proportions).

Like in men, mean age, comorbidity and prevalence of smoking, hypertension and atrial fibrillation have also raised along the study period in women with and without diabetes (Table 2).

If we compare diabetic men with diabetic women we find that men have higher crude estimated incidence than women in all years analyzed.

Men are significantly younger (71.57 vs 77.51 years), have higher CCI (CCI 2, 42.88% vs 38.29%) and smoke (33.57% vs 2.84%) in greater proportion than women along the study period. On the other hand women show more atrial fibrillation (30.14% vs 17.56%) and hypertension (70.57%vs 63.17%) than men.

Tables 3 and 4 show the diagnostic and therapeutic procedures and in-hospital outcomes for men and women with an ischemic stroke according to diabetes status over the period of study.

**Table 3:** Hospitalization outcomes and therapeutic and diagnostic procedures for hospital discharges due to ischemic stroke among men with and without type 2 diabetes in Spain, 2003–2012.

	2003	2004	2005	2006	2007	2008	2009	2010	2011	2012	Total
NO DIABETES											
IHM, n (%)*	1369(11.26)	1328(10.76)	1394(11.18)	1630(11.02)	1752(11.13)	1780(10.66)	1784(10.6)	1852(10.71)	1727(9.98)	1714(9.78)	16330(10.66)
LOHS, median (IQR)*	8(9)	8(8)	8(8)	8(8)	8(8)	8(8)	8(8)	7(7)	7(8)	7(7)	8(8)
Readmission, n(%)	630(5.18)	648(5.25)	571(4.58)	718(4.85)	785(4.99)	835(5)	829(4.93)	873(5.05)	857(4.95)	898(5.12)	7644(4.99)
Fibrinolysis, n (%)*	64(0.53)	102(0.83)	137(1.1)	279(1.89)	358(2.27)	521(3.12)	750(4.46)	1032(5.97)	1209(6.99)	1421(8.11)	5873(3.83)
CAT, n (%)*	9253(76.1)	9312(75.43)	9579(76.81)	11233(75.94)	11939(75.85)	13124(78.62)	13267(78.84)	13681(79.11)	13768(79.55)	14181(80.92)	119337(77.92)
Magnetic resonance, n (%)*	2652(21.81)	3084(24.98)	3271(26.23)	3910(26.43)	4451(28.28)	5361(32.12)	5747(34.15)	6071(35.11)	6090(35.19)	6017(34.34)	46654(30.46)
Doppler echocardiography, n (%)*	3393(27.91)	3653(29.59)	3961(31.76)	4538(30.68)	4858(30.86)	5809(34.8)	6446(38.31)	6716(38.84)	6857(39.62)	7079(40.4)	53310(34.81)
Mechanical ventilation, n (%)*	216(1.78)	182(1.47)	213(1.71)	244(1.65)	273(1.73)	325(1.95)	330(1.96)	356(2.06)	388(2.24)	370(2.11)	2897(1.89)
Parenteral nutrition, n (%)*	205(1.69)	235(1.9)	271(2.17)	257(1.74)	392(2.49)	419(2.51)	439(2.61)	501(2.9)	471(2.72)	455(2.6)	3645(2.38)
DIABETES											
IHM, n (%)	485(10.17)	473(9.31)	535(9.95)	657(10.56)	705(10.08)	729(9.63)	744(9.68)	736(9.28)	765(9.25)	781(9.33)	6610(9.68)
LOHS, median (IQR)*	9(9)	8(9)	8(8)	8(8)	8(8)	8(8)	8(8)	8(7)	7(8)	7(7)	8(8)
Readmission, n (%)	247(5.18)	260(5.12)	303(5.64)	385(6.19)	404(5.78)	427(5.64)	460(5.99)	462(5.82)	499(6.04)	521(6.23)	3968(5.81)
Fibrinolysis, n (%)*	13(0.27)	21(0.41)	32(0.6)	62(1)	98(1.4)	112(1.48)	214(2.79)	315(3.97)	377(4.56)	410(4.9)	1654(2.42)
CAT, n (%)*	3690(77.34)	3847(75.73)	4253(79.13)	4759(76.5)	5411(77.36)	6056(79.98)	6177(80.39)	6427(81.01)	6710(81.16)	6845(81.81)	54175(79.36)
Magnetic resonance, n (%)*	1069(22.41)	1284(25.28)	1470(27.35)	1736(27.91)	2076(29.68)	2440(32.22)	2612(33.99)	2752(34.69)	2993(36.2)	2931(35.03)	21363(31.29)
Doppler echocardiography, n (%)*	1392(29.18)	1479(29.11)	1791(33.32)	1980(31.83)	2207(31.55)	2629(34.72)	2986(38.86)	3168(39.93)	3314(40.08)	3435(41.05)	24381(35.71)
Mechanical ventilation, n (%)	59(1.24)	68(1.34)	52(0.97)	86(1.38)	95(1.36)	102(1.35)	104(1.35)	118(1.49)	117(1.42)	144(1.72)	945(1.38)
Parenteral nutrition, n (%)	70(1.47)	86(1.69)	112(2.08)	93(1.49)	162(2.32)	189(2.5)	192(2.5)	241(3.04)	191(2.31)	200(2.39)	1536(2.25)

LOHS: length of stay; IHM: In-hospital mortality; CAT: Computed tomography angiography.

* P<0.05 (Comparison by year: Binary logistic regression for incidence, Kruskal-Wallis for medians, Pearson´s chi-square for proportions).

**Table 4:** Hospitalization outcomes and therapeutic and diagnostic procedures for hospital discharges due to ischemic stroke among women with and without type 2 diabetes in Spain, 2003–2012.

	2003	2004	2005	2006	2007	2008	2009	2010	2011	2012	Total
NO DIABETES											
IHM, n (%)	1575(14.86)	1474(13.89)	1609(14.71)	1926(14.68)	2028(14.19)	2171(14.36)	2216(14.22)	2262(14.07)	2269(14.04)	2324(13.7)	19854(14.24)
LOHS, median (IQR)*	9(10)	9(9)	8(9)	8(9)	8(8)	8(9)	8(8)	8(9)	7(8)	7(8)	8(8)
Readmission, n (%)*	508(4.79)	506(4.77)	500(4.57)	724(5.52)	725(5.07)	787(5.21)	810(5.2)	832(5.18)	866(5.36)	907(5.35)	7165(5.14)
Fibrinolysis, n (%)*	46(0.43)	62(0.58)	124(1.13)	201(1.53)	251(1.76)	369(2.44)	650(4.17)	867(5.39)	983(6.08)	1271(7.49)	4824(3.46)
CAT, n (%)*	8067(76.13)	8045(75.83)	8495(77.64)	9982(76.07)	10871(76.04)	11759(77.8)	12306(78.96)	12618(78.48)	12835(79.42)	13757(81.09)	108735(77.97)
Magnetic resonance, n (%)*	1587(14.98)	1746(16.46)	1929(17.63)	2327(17.73)	2811(19.66)	3235(21.4)	3554(22.8)	3788(23.56)	4026(24.91)	4025(23.73)	29028(20.81)
Doppler echocardiography, n (%)*	2201(20.77)	2315(21.82)	2628(24.02)	3195(24.35)	3415(23.89)	3955(26.17)	4631(29.71)	4948(30.78)	4951(30.64)	5397(31.81)	37636(26.99)
Mechanical ventilation, n (%)*	121(1.14)	108(1.02)	117(1.07)	129(0.98)	146(1.02)	156(1.03)	193(1.24)	203(1.26)	207(1.28)	226(1.33)	1606(1.15)
Parenteral nutrition, n (%)*	325(3.07)	358(3.37)	410(3.75)	437(3.33)	512(3.58)	589(3.9)	695(4.46)	726(4.52)	707(4.38)	680(4.01)	5439(3.9)
DIABETES											
IHM, n (%)	628(13.21)	636(13.26)	752(14.86)	817(13.72)	917(14.76)	986(14.25)	980(13.52)	1025(14.32)	1000(13.83)	1030(14.2)	8771(14.01)
LOHS, median (IQR)*	9(10)	9(10)	9(9.5)	9(9)	9(9)	8(9)	8(9)	8(8)	8(9)	7(8)	8(9)
Readmission, n (%)*	287(6.04)	296(6.17)	322(6.36)	366(6.15)	417(6.71)	506(7.31)	483(6.66)	507(7.08)	509(7.04)	503(6.93)	4196(6.7)
Fibrinolysis, n (%)*	9(0.19)	10(0.21)	34(0.67)	34(0.57)	71(1.14)	82(1.19)	170(2.34)	266(3.72)	307(4.25)	326(4.49)	1309(2.09)
CAT, n (%)*	3723(78.3)	3720(77.53)	4093(80.89)	4686(78.72)	4857(78.16)	5531(79.95)	5870(80.95)	5710(79.79)	5947(82.24)	6034(83.17)	50171(80.16)
Magnetic resonance, n (%)*	792(16.66)	763(15.9)	911(18)	1029(17.29)	1196(19.25)	1479(21.38)	1676(23.11)	1694(23.67)	1730(23.92)	1721(23.72)	12991(20.76)
Doppler echocardiography, n (%)*	1060(22.29)	1078(22.47)	1236(24.43)	1462(24.56)	1489(23.96)	1839(26.58)	2188(30.18)	2183(30.51)	2186(30.23)	2317(31.94)	17038(27.22)
Mechanical ventilation, n (%)*	39(0.82)	44(0.92)	47(0.93)	38(0.64)	56(0.9)	54(0.78)	56(0.77)	67(0.94)	69(0.95)	81(1.12)	551(0.88)
Parenteral nutrition, n (%)*	162(3.41)	161(3.36)	177(3.5)	209(3.51)	214(3.44)	290(4.19)	358(4.94)	291(4.07)	320(4.43)	286(3.94)	2468(3.94)

LOHS: length of stay; IHM: In-hospital mortality; CAT: Computed tomography angiography.

* P<0.05 (Comparison by year: Binary logistic regression for incidence, Kruskal-Wallis for medians, Pearson´s chi-square for proportions)

The IHM among men with type 2 diabetes did not change significantly over the period of study ranging from 10.56% to 9.25%. Crude estimates of IHM were higher among those without diabetes, but significantly decreased along the period of analysis (11.26% in 2003 to 9.78% in 2012) (Table 3).

The mean LOHS fell significantly from 9 days in 2003 to 7 days in 2012 in diabetic men and in those without diabetes from 8 days in 2003 to 7 days in 2012 (p<0.05). Readmission remained stable over time for men (both groups) with figures around 5% in all the years studied.

As can been seen in Table 3, a significant increase in the use of fibrinolysis was found raising from 0.27% in 2003 to 4.9% in 2012 was among diabetic men and from 0.53% to 8.1% among those without the disease.

Of the diagnostic procedures analyzed the most commonly used was CAT followed by Doppler echocardiography and magnetic resonance. The use of all diagnostic procedures have increased in the last ten years in diabetic and non-diabetic men with similar figures for both groups.

The IHM among women with an ischemic stroke did not change significantly for those with and without type 2 diabetes, as can we see in Table 4, with similar crude figures for both groups of around 13–14%. Over the 10-year study period, LOHS in women with and without diabetes decreased significantly from 9 days (IQR, 10 days) in 2003 to 7 days (IQR, 8 days) in 2012. We found an slightly but significant increase in the frequency of readmission for women over the period of study (6.04% in 2003 to 6.93% in 2012 in women with type 2 diabetes and 4.79% in 2003 to 5.35% in 2012 in women without diabetes).

As observed for men a significant increase in the use of fibrinolysis from 0.19% to 4.49% in diabetic women and from 0.43% to 7.49% in non-diabetic women with an ischemic stroke was found along the study period. The most commonly used diagnostic procedure was CAT for both groups of women. However it was used in a higher proportion among those with than without diabetes in all the years studied. The use of the three diagnostic procedures analyzed has increased in diabetic and non-diabetic women over time.

When we compared hospitalization outcomes by gender, we found that crude IHM estimates were always higher in woman than men in diabetic group as well as non diabetic group, and for every year considered. Readmission rates were also higher among women in the total study population and for all the years analyzed. The use of CAT was similar for both sexes but magnetic resonance (31.29% vs 20.76%) and Doppler echocardiography (35.71% vs. 27.22%) were used in a significantly higher proportion of diabetic men than diabetic women.

The Poisson regression models conducted to assess the adjusted time trend in the incidence rate of ischemic stroke hospitalization from 2003 to 2012 in Spain, yielded an adjusted incidence rate ratio (IRR) for men with type 2 diabetes of 1.02 (95%CI 1.01–1.03). This result shows that after adjusting for possible confounders the incidence has increases, on average, 2% per year.

The IRR for diabetic women was also 1.02 (95%CI 1.01–1.03) so the average increase per year was identical for both sexes. We also found this positive time trend among men and women without diabetes (IRR 1.03 95% CI 1.02–1.04 for both sexes).

We merged data subsets of men with and without diabetes, to assess the effect of the disease in the incidence of ischemic stroke hospitalizations, we found an IRR of 2.27 (95%CI 2.24–2.29). The corresponding figure for women was 2.15 (95%CI 2.13–2.17). This means that after adjusting for possible confounders the incidence among diabetic men and women were 2.27 and 2.15 times higher than among non diabetic men and women respectively (IR for interaction term sex by diabetes 1.044[1.031–1.058] (p<0.001).

Table 5 summarize the results of multivariate logistic regression analysis for factors associated with IHM among men and women with and without diabetes hospitalized for an ischemic stroke. Figures are Odds Ratio with 95% confidence interval.

**Table 5:** Multivariate binary logistic regression analysis of the factors associated with in hospital mortality after ischemic stroke hospitalization among men and women with and without type 2 diabetes in Spain, 2003–2012.

	Men			Women		
Age (years)	No Diabetes	Diabetes	Total	No Diabetes	Diabetes	Total
15–44	1	1	1	1	1	1
45–54	1.33(1.12–1.58)	1.32(0.75–2.32)	1.26(1.07–1.47)	1.51(1.23–1.87)	1.20(0.60–2.39)	1.43(1.18–1.75)
55–64	1.58(1.35–1.85)	1.88(1.09–3.22))	1.53(1.31–1.77)	1.68(1.38–2.03)	1.39(0.73–2.66)	1.57(1.30–1.88)
65–74	2.19(1.88–2.54)	2.88(1.69–4.90	2.18(1.89–2.52)	2.08(1.73–2.49)	1.81(0.96–3.44)	1.97(1.66–2.34)
75–84	3.41(2.94–3.95)	4.59(2.69–7.83)	3.42(2.97–3.94)	3.37(2.82–4.02)	3.13(1.66–5.91)	3.27(2.76–3.87)
>84	6.38(5.50–7.42)	8.35(4.88–14.26)	6.36(5.51–7.35)	6.80(5.70–8.11)	5.75(3.04–10.88)	6.42(5.42–7.59)
CCI						
0–1	1	1	1	1	1	1
2	1.44(1.39–1.49)	1.53(1.45–1.61)	1.46(1.42–1.50)	1.24(1.20–1.28)	1.38(1.32–1.45)	1.28(1.25–1.31)
≥ 3	2.41(2.20–2.64)	2.73(2.38–3.14)	2.49(2.30–2.69)	1.87(1.65–2.11)	2.24(1.89–2.65)	1.97(1.78–2.18)
Atrial Fibrillation						
No	1	1	1	1	1	1
Yes	1.36(1.31–1.42)	1.73(1.63–1.83)	1.46(1.41–1.50)	1.37(1.33–1.42)	1.67(1.59–1.75)	1.45(1.42–1.49)
Readmission						
No	1	1	1	1	1	1
Yes	1.80(1.70–1.92)	1.82(1.66–1.98)	1.81(1.72–1.90)	1.81(1.70–1.91)	1.79(1.65–1.93)	1.80(1.72–1.89)
Fibrinolysis						
No	1	1	1	1	1	1
Yes	1.32(1.21–1.44)	1.65(1.42–1.92)	1.39(1.29–1.50)	0.94(0.86–1.04)	1.16(0.98–1.36)	0.99(0.91–1.08)
Year	0.97 (0.96–0.97)	0.96(0.95–0.97)	0.97(0.96–0.98)	0.98(0.97–0.99)	0.98(0.96–0.99)	0.98(0.97–0.99)
Diabetes						
No			1			1
Yes			0.98(0.95–1.02)			1.07(1.05–1.11)

CCI (Charlson Comorbidity Index): Comorbidities included in the Charlson comorbidity index, except diabetes and stroke.

Figures are Odds Ratios (95% CI)

Among men and women with diabetes, IHM was significantly greater in older subjects (OR 8.35; 95%CI 4.88–14.26 and OR 5.75;95%CI 3.04–10.88 in ≥84 aged group compared with reference category 15–44 years, respectively), in those with more comorbidities (OR 2.73; 95%CI, 2.38–3.14 and OR 2.24; 95%CI, 1.89–2.65 for those with ≥3 comorbidities, respectively) and in those with atrial fibrillation (OR 1.73; 95%CI, 1.63–1.83 and OR 1.67;95%CI 1.59–1.75, respectively). Those diabetic men and women who received a fibrinolysis procedure had higher probability of dying (1.65-fold and 1.16-fold, respectively) during their stay than those who did not undergo this procedure.

Over the entire period of time and after adjusting the model for the rest of the variables, those diabetic men and women who had a readmission also had higher probability of dying (1.82 times and 1.79 times) during their stay than those men and women without readmission for ischemic stroke. Time trend analysis showed a significant decrease in IHM from 2003 to 2012 in diabetic men and women (OR 0.96 95% CI 0.95–0.97 and OR 0.98; 95%CI 0.96–0.99). As can be seen in Table 5, same variables were associated to IHM among those men and women without diabetes. When we analyzed the entire database, women with type 2 diabetes had significantly higher mortality than women without diabetes after adjusting for all covariates (OR, 1.07; 95%CI, 1.05–1.11) and no difference was found for men.

## Discussion

In this observational, retrospective, national-based study of more than 400,000 events, we have found that the incidence of discharge due to ischemic stroke in Spain from 2003 to 2012 has increased in all groups. After adjusting for possible confounders, the incidence has increased at a mean of 2% per year in diabetic and 3% in non diabetic patients.

Our results contrast with the decline on the incidence described in the last few decades in our country [24] and other international series [25,26] attributed to the measures adopted to control risk factors. Similarly, in Greece, Vasiliadis et al reports a decline of almost 20% despite the projection on stroke incidence as a result of economic crisis [27]. It is worth noting that the mentioned Greek study finished in 2002 and the impact of economic crisis could be more important in recent years. Stroke mortality has been gradually declining in Spain. From 1990 to 2006 stroke mortality decrease 2% per year on average [28]. Cayuela et al. [29] showed decreases even larger (-6.3% for women and -7.2% for men) on mortality rates for the period 2005–2011. Lower mortality rates (higher survival rates) would make compatible an increase in the incidence of hospitalization (first and subsequent events) due to stroke with the aforementioned decrease on stroke incidence.

As expected we found that incidence rate of hospitalization due to ischemic stroke were higher among those with than those without diabetes in all the years studied [30]. Diabetes is often associated with a number of diseases and medical conditions such as hypertension, obesity, dyslipidemia, hyperglycemia and inflammation that accelerate and aggravate the atherosclerotic process and thus favor the onset of stroke [31]. After adjusting for potential confounders, the independent effect of diabetes on the incidence of ischemic stroke hospitalization remained significant for both sexes accordingly with literature [5,32].

People admitted to hospital due to ischemic stroke were progressively older. This was expected because age is one of the risk factors more strongly associated to stroke [10]. For both groups studied, risk factors associated to stroke such as hypertension, smoking and the prevalence of atrial fibrillation showed a significant increase along the study period. This is in concordance with previous studies which highlight that stroke patients are far from optimal control of cardiovascular risk factors in our country. According National Health Survey, overall smoking prevalence in general population have decreased in Spain along the period analyzed (2004–2011), but this decrease is mainly due to population under 45. For the same period, prevalence of smoking has increased in people over 55, so a higher prevalence of smoking was expected.

Similarly prevalence of hypertension in Spain has been traditionally higher than in neighboring countries [33]. and National Health Survey show an increase on the prevalence of self-declared hypertension for all age groups. Arboix et al also found an increase in the prevalence of HBP among first-stroke patients in the Stroke Registry of Barcelona[34]. Furthermore, ICTUSCARE study, that included 975 patients in secondary prevention, showed that only 17.6% of them achieved recommended values of blood pressure [35]

Since diabetes is a risk factor for AF, we expected a higher prevalence in this group than among non diabetic ones. A plausible explanation to this unexpected finding is that diabetic patients suffer more frequently lacunar and atherothrombotic strokes due to atherosclerosis rather than cardioembolic ones associated to AF[36]

As described in general population, we have found a higher incidence in men than in women in both diabetic and non-diabetic patients except for older than 85 years. Men have a higher age-specific stroke incidence rate of hospitalization except for women aged 35–44 years (probably related to pregnancy and the use of contraceptives) and those older than 85 years. Their relative longevity contributes to the higher risk of stroke in older women [37].

In our study, men were younger, more often were smokers and had more comorbidities. There is a recognized group of younger patients, mostly men <65 years, which has cardiovascular risk factors undiagnosed or uncontrolled [38]. Efforts to identify risk factors and implement preventive and therapeutic measures in this subgroup are needed.

Sex differences in the association of T2DM with stroke has been reported in previous studies with conflicting results. The meta-analysis of Peters shows an excess risk of stroke significantly higher in women than in men independent of other major cardiovascular risk factors After adjustment, the relative risk of stroke associated with diabetes was 2.28 (95% CI 1.93–2.69) for women and 1.83 (1.60–2.08) for men, and the ratio of relative risks between women and men with diabetes was 1.27 (1.10–1.46) [39]. The findings of Shah et al in a cohort study of nearly 2 million people are not consistent with this previous report. They didn´t find gender differences in associations between T2DM and cardiovascular outcomes except for incident coronary heart disease [8]. In our study we noted weak evidence of a slightly stronger association of T2DM with ischemic stroke in men than in women, IRR of 2.27 for men (95%CI 2.24–2.29) vs 2.15 (95%CI 2.13–2.17) for women. Nevertheless, diabetic women had higher crude IHM and readmission rates than diabetic men consistently with previous evidence. Investigators of the UK Prospective Diabetes Study reported that women with diabetes had more than twice the risk of men with diabetes for stroke case fatality [40]. The poorer survival after stroke of women with diabetes compared with men was also noted in the MONICA study [7]. The mechanism involved could rely on gender differences in diabetes-related changes in the progression of atherosclerosis or in more novel risk factors (such as markers of coagulation and inflammation, lipid peroxidation, and endothelial function [41–43] Mansfield and colleagues reported that women had significantly higher factor VII and plasminogen activator inhibitor 1 activity than men [44]. Similarly, data from the British Regional Heart Study and the British Women’s Heart Health Study showed a greater adverse effect of diabetes in women than in men on markers of coagulation, fibrinolysis, lipids, and blood pressure, which were mediated by greater changes in central adiposity and insulin resistance in women [45]. Further work is needed to clarify whether the excess risk of stroke conferred by diabetes differs between the sexes and the mechanism involved.

In-hospital mortality has remained stable over the study period among diabetic patients and non diabetic women whereas it has decreased in the group of non-diabetic men. Older patients, higher coexisting comorbidities and atrial fibrillation were the variable associated to IHM for all groups. Comorbidity has been associated with stroke mortality and poor outcomes in a previous study conducted in our country [46]. Age is also a well-known non modifiable risk factor for stroke mortality. Not surprisingly, we have found that mortality among diabetic patients was significantly higher in the very elderly (> 84 years). On the other hand, after adjusting for covariates (Age-groups, CCI, AF and fibrinolysis) diabetes slightly increase the risk of IHM among women (1.07[1.05–1.11]) but not among men (0.98[0.95–1.02]). Influence of diabetes has been previously described to be time dependent in men. In the first 30 days after incident stroke, mortality was lower in diabetic than in nondiabetic individuals [47]

We also observed a reduction in LOHS in this period. However with this dataset we are not able to ascertain the reason for this minor reduction in the LOHS. Budget restrictions could influence this figure in the period 2008–2012 but a better clinical management of patients cannot be discarded either.

The management of patients admitted for ischemic stroke has included more often effective therapeutic procedures as fibrinolysis. Our study shows that there has been an enormous increase (over seven fold for every subgroup after stratifying by gender and diabetes) in the use of fibrinolysis from 2003 to 2012 accordingly with guidelines recommendations. Intravenous thrombolysis with recombinant tissue-type plasminogen activator (r-tPA), alteplase, improves survival free of dependency for ≤18 months after stroke, despite the risk of early symptomatic intracranial hemorrhage (sICH) [48]. Fibrinolysis increases the odds of ICH by ≈4-fold and large or symptomatic ICH that are not immediately fatal are associated with an increased risk of subsequent death or disability [49,50].

In our study those diabetic men and women who underwent fibrinolysis had higher probability of dying. This could be related to a higher risk of hemorrhage although it´s only a hypothesis because we lack this information due to the nature of our study. Higher severity increase the chance of fibrinolysis indication and at the same time is related to the risk of death. But other baseline features usually weighed up when considering endovascular treatment like 1) time since onset of ictus, 2) severity of neurologic deficit, 3) age 4) baseline functional status 5) information suggestive of stroke etiology and 6) vascular anatomy [51] can also play similar role.

Our analysis only include ischemic stroke. We also include age group as a covariate but we don’t have information about either time since onset of ictus, severity, baseline functional status or anatomical localization. So we cannot evaluate the role of this variables on IHM. It has been suggested that diabetic people and patients with history of prior stroke could have an unacceptable high risk of hemorrhagic transformation [52] but there have been published conflicting results. Zhang et al recently published that diabetes is significantly associated to hemorrhagic transformation after thrombolysis [53] while Lindley et al report the outcomes by clinically important subgroups in the Third International Stroke Trial and they did not identify any subgroups for whom treatment should be avoided, including diabetic patients [54]. It´s worth mentioning that only 3035 of the originally planned 6000 patients were recruited, and the trial was underpowered for the primary outcome.

The strength of our investigation lies in its large sample size, its 10-year follow-up period and its standardized methodology, which has been with previously used to investigate diabetes and its complications in Spain and elsewhere [55]. Nevertheless, our study is subject to a series of limitations. Our data source was the MBDS. This database contains administrative discharge data for Spanish hospitalizations and uses information the physician included in the discharge report. As we described in the methods section, readmission variable (“New hospitalization within 30 days after discharge.”) is created before database is anonymize prior to delivery for research purposes, so that we are not able to distinguish first from subsequent events. Furthermore if stroke hospitalization occur after 30 days, the event is indistinguishable from first event of any other patient. Anyway, previous studies with MBDS showed readmission only account for 7% of the stroke hospitalizations [56], so that readmission will have a minor impact on our estimates.

We are not able to identify which of these patients died due to a hemorrhagic transformation after fibrinolysis or if they were under anticoagulation or antiplatelet therapy. Another significant limitation is the fact that we have not broken down our diabetic patients into groups based on therapy used to controlling blood glucose, and we were unable to state their glycosylated hemoglobin.

We are aware that considering other databases, such as mortality registries, beside discharge data would be advisable in order to detect patients with ischemic stroke dying outside hospital. Unfortunately in Spain this process is still unavailable for us.

In conclusion our national data show that incidence of ischemic stroke increased significantly during the period of study (2003–2012). People with T2DM have more than double the risk of ischemic stroke after adjusting for other risk factors. For diabetic and non diabetic patients the use of fibrinolysis has increased over time and it was associated with increased mortality in patients with T2DM. Women with T2DM had poorer outcomes- IHM and readmission rates- than diabetic men. Diabetes was an independent factor for IHM only in women.

## Abbreviations

CAT: Computed tomography angiography
CCI: Charlson comorbidity index
CVA: Cerebrovascular accidents
CMBD: Spanish Minimum Basic Data Set (Conjunto Mínimo Básico de Datos)
ICD-9-CM: International Classification Diseases-Ninth Revision, Clinical Modification
ICH: Intracranial hemorrhage
IHM: In-hospital mortality
IRR: Incidence rate ratio
LOHS: Length of hospital stay
OR: Odds ratio
SD: Standard deviation
T2DM: Type 2 diabetes
