# Correction: Time Trends in Ischemic Stroke among Type 2 Diabetic and Non-Diabetic Patients: Analysis of the Spanish National Hospital Discharge Data (2003-2012)

**DOI:** 10.1371/journal.pone.0160476

**Published:** 2016-07-28

**Authors:** Nuria Muñoz-Rivas, Manuel Méndez-Bailón, Valentín Hernández-Barrera, José Ma de Miguel-Yanes, Rodrigo Jiménez-García, Jesús Esteban-Hernández, Isabel Jiménez-Trujillo, Alejandro Alvaro-Meca, Pilar Carrasco-Garrido, Javier de Miguel-Díez, Ana López-de-Andrés

There are errors in the Funding section. The correct funding information is as follows: This study was funded by the FIS (Fondo de Investigaciones Sanitarias—Health Research Fund, grant no. PI13/00118, Instituto de Salud Carlos III) co-financed by the European Union through the Fondo Europeo de Desarrollo Regional (FEDER, “Una manera de hacer Europa”), and by the Grupo de Excelencia Investigadora URJC-Banco Santander No. 30VCPIGI03: Investigación traslacional en el proceso de salud—enfermedad (ITPSE). The funders had no role in study design, data collection and analysis, decision to publish, or preparation of the manuscript.
